# A Treatise upon the Electro-Physiological Phenomena of Animals

**Published:** 1845-04

**Authors:** 


### MEDICO-CHIRURGICAL

### REVIEW.

---

APRIL, 1845.

---

I. Traité des Phénomenes Electro-physiologiques des Animaux. Par *C. Matteucci.* Suivi d'Etudes Anatomiques sur le Systeme Nerveux et sur l'Organe Electrique de la Torpille. Par *Paul Savi.* Paris, 1844. Octavo, pp. 348. Plates.

A Treatise upon the Electro-physiological Phenomena of Animals. By *C. Matteucci.* To which are added, Anatomical Investigations respecting the Nervous System, and the Electric Organ of the Torpedo. By *Paul Savi.*

II. Rapport entre le sens du Courant Electrique et les Contractions Musculaires dues a ce Courant. Par MM. *Longet* et *Ch. Matteucci.* (Comptes rendres de l'Académie des Sciences, du 9 Septembre, 1844).

On the Connexion between the Direction of the Electric Current and the Muscular Contractions excited by that Current. By MM. *Longet* and *Ch. Matteucci.*

Few subjects present more interesting topics of inquiry to the physiologist, than the connexion between the vital and inorganic forces. For nearly half a century physiological writers have thrown out sundry vague suggestions or opinions of the possibility or even probability of the existence of some mysterious connexion between electricity and vitality. Of the precise nature of that connexion they themselves had no definite idea, and, therefore, were unable to express themselves otherwise than in general, vague, and often in unmeaning terms. Abernethy, who adopted Hunter's opinion, that there is a principle of life independent of organization—a something superadded to organised structure—boldly stated his belief that the vital principle was either electricity or something of a similar nature. On this view, then, it is assumed, that every vital process in the animal body, nay, in the vegetable also, is attributable to electric action;—an extravagant assumption hitherto unsupported by facts.

With some few exceptions, later writers have been content to take a more limited view of the connexion between electricity and the animal functions, and have ascribed some only of the vital processes to electrical agency. Secretion and nervous action have formed the most favorite processes for explanation by electro-physiological hypotheses. The resolution of neutral salts into their acid and basic constituents by electricity led Dr. Wollaston, about thirty-six years since, to throw out the suggestion that possibly the products of secretion might be owing to electricity of low tension. The actual existence of certain analogies between electricity and the active principle of the nerves, and the supposed existence of others, have induced many physiologists to regard the nervous cords as electrical conductors, and nervous action as identical with electrical action. To test the accuracy of this hypothesis, endeavours have been from time to time made to detect electric currents in the nerves, but in no instance with satisfactory results. In some cases, indeed, these attempts have been reported as successful; but, when the experiments have been repeated by those most competent to make them, and with the requisite precautions to guard against all known sources of fallacy, the result has hitherto, in every instance, been a failure; and we believe that, up to the present time, there are no well-grounded facts on which we can venture to assert the existence of an electric current in the nerves; or to contend for the identity of nervous and electric action. Indeed, as a recent able physiological writer (Müller) has justly observed, though we may be able to generate electric action in the nerves, yet the mode of action of the nerves is wholly different from that of electricity.

Matteucci has been, for several years, favorably known to the scientific world by his researches in Electro-physiology. Those of his investigations which form the subject of the present notice, and which are contained in the book and paper whose titles are placed at the head of this article, are of a highly interesting nature. They derive additional importance from the fact, that the Council of the Royal Society, on the recommendation of its Committee of Physics (which includes Messrs. Faraday, Daniell, Wheatsone, and other distinguished electricians), has recently adjudged the Copley Medal to Matteucci, for his novel, ingenious, and important electro-physiological discoveries. During the last Autumn, Matteucci came to this country to attend the meeting of the British Association, and demonstrated the most important of his results, on the electricity of animals, to a considerable number of persons, both in London and elsewhere. The dexterity and facility with which he conducted his experiments, the very marked and satisfactory results which he obtained, and the numerous precautions which he employed to guard against fallacies, cannot be too highly praised. Every one who witnessed his demonstrations felt satisfied as to the reality and constancy of the effects which he saw, and the experiments have since been repeated by others (including ourselves) with the most satisfactory results.

In our personal communications with Matteucci, as well as in the perusal of his writings, we have been struck and delighted with the remarkable candour and caution which he has everywhere exhibited. He seems to have no desire to ascribe to his subject more importance than it really deserves; no wish to give the power of which he treats a more general or universal agency than it actually possesses. Instead of regarding the vital functions as resulting from electrical action, as some previous writers have done, he reverses the picture, and shows us that the electrical currents, which he has detected in the animal body, are not the causes, but the effects, of the changes going on in the living machine. He repudiates the idea of the identity of the nervous force with electricity. He tells us that he has in vain sought for an electric current in the nerves, and, further, that the properties and laws of the propagation of electricity are incompatible with the idea of such a current.

In noticing Matteucci's works, we do not think it necessary to examine all the subjects which he has investigated, or to follow the precise order which he has done, in placing before our readers what we conceive to be the more novel and important, and, therefore, the most interesting, of the results which he has obtained.

1. In the *Historical Induction* to his work, Matteucci corrects several popular errors which are prevalent with respect to the discovery of Galvanism. He informs us, on the authority of Duméril, that Swammerdam, in his *Biblia Naturæ*, vol. ii., p. 849, is the earliest writer who is known to have observed contractions excited in the muscles of an animal by a voltaic combination. The precise form of the apparatus which was used is not, however, very clearly described: but it consisted of silver and copper. The experiment is, in fact, essentially the same as one subsequently made by Galvani; who does not, however, appear to have been acquainted with that made by Swammerdam, notwithstanding that it was performed before the Grand Duke of Tuscany.

Our readers will no doubt be surprised to find that the story told, in almost every treatise on Electricity, of the accidental discovery of Galvanism is incorrect. Alibert, in his *Eloge* on Galvani, contained in the 4th volume of the *Memoires de la Sociëté Médicale d'Emulation*, and Chaumeton, in the *Biographie Universelle*, give very interesting details respecting the accidental observation by Madame Galvani, of the convulsions excited in frogs (prepared for her dietetical use), by the electrical spark. These details, it appears, are fabulous, for Matteucci states that—

"The Institute of Bologna possesses some unexceptionable documents which fix the period at which Galvani commenced his first experiments on the frog. In the register signed by the celebrated Canterzani, Secretary to the Academy, are the following notes relative to the dates of the memoirs which Galvani had communicated:

April 9th, 1772. *On Hallerian Irritability.*
April 20th, 1773. *On the Muscular Movements of Frogs.*
Jan. 20th, 1774. *On the Action of Opium on the Nerves of Frogs.*

So that twenty-one years before the publication of the celebrated commentary, *De Viribus Electricitalibus*, and made in 1791, Galvani had commenced his experiments on the contractions of frogs: and notwithstanding that he was most completely devoted to his wife, we must not believe what Alibert states in his *Eloge*, viz. that the frogs, in which Galvani saw the first contractions produced by electric action, had been accidentally placed on the table by the side of the electric machine, and were intended to be employed in the preparation of soup for his Lucy. Among the manuscripts of Galvani has also been found the journal in which is contained a description of his first experiments on the contractions excited in frogs by what he calls artificial electricity. It is dated November 6th, 1780—that is, eleven years before the publication of his Commentary—and in his first experiment he writes, '*The frog was prepared in the usual manner,*' *(alla solita maniera.)* Evidently, therefore, it was not the first time that he had made experiments on frogs." 6.

A. We must content ourselves with one more extract from the historical part of Matteucci's *Traité*, and then proceed to other topics.

" The first phenomenon which Galvani describes in his Commentary is that which has been criticised by all historians, and which, according to them, proves that *he was entirely ignorant of the true theory of electrical influences.* We know that Galvani observed the contraction of a frog prepared in the ordinary way, *de more parata*, every time that, being in communication with the floor, by a conducting substance, a spark was drawn from the conductor of the machine, more or less near to which was the frog.

" Nothing can be more unjust than this criticism. Galvani was surprised at the effect described, and so would have been, at that period, any philosopher. Galvani studied the phenomenon with every care and the greatest sagacity. Moreover, in a Latin memoir, which is very little known, and in which he treats of the electrical light observed in air more or less rarefied, it is easy to perceive that Galvani was quite up to all the discoveries and theories of electricity of that period. In his memoir, *On the Employment and Activity of the Conducting Arc*, he says that the contraction of the frog may be very well explained, in the case above-mentioned, by the returning stroke *(le coup de retour.)* So that he explained the phenomenon as we now do.

" In pursuing his researches, Galvani submitted the prepared frog to the passage of atmospheric electricity; and it was in performing these experiments that he discovered that the frog was the most delicate of all electroscopes. We cannot read, without shuddering, the details of an experiment described in his journal, and made on the 7th of April, 1786. Galvani held in his hands the insulated atmospheric conductor at the instant of the occurrence of a loud clap of thunder: the experiment of the metallic arc was yet to be made!" 7.

B. We much regret that our space does not permit us to enter into further details respecting Galvani's discoveries and theories; which never appear to us to have been appreciated as they have deserved, and which were eclipsed by the subsequent splendid discoveries of Volta. To Matteucci is due the credit of re-directing public attention to them. After reading his work we rose with a much augmented veneration for the genius, the industry, and the sagacity of Galvani; and we look forward with much interest to the appearance of the entire works of Galvani, which are preparing for publication under the auspices of the Academy of Sciences of Bologna.

2. *Instruments employed in Electro-physiological Researches.*—Matteucci describes five instruments which are employed in experiments on electrical physiology. They are as follows: 1st. *Nobili's Galvanometer with the Astatic Needle.* [A figure and description of this instrument are contained in the second volume (p. 504) of Lardner and Walker's *Manual of Electricity*, (Lardner's Cyclopædia.)] *Rumkorf's condenser* (composed of a pair of box magnets) has been contrived to augment the sensibility of the galvanometer. 2ndly. *The Frog Galvanoscope*, which will be described presently. 3rdly. *The Electroscope*, (with or without Volta's condenser,) for detecting tension electricity. 4thly. *A Constant Battery*, composed of plates of copper and of amalgamated zinc. The copper plate is immersed in a solution of sulphate of copper; while the zinc plate is excited by a solution of common salt or by water acidulated with sulphuric acid. 5thly. *A Tangential Needle,* (boussole de tangente) to ascertain the constancy of the current obtained by the pile.

The *Frog Galvanoscope* is an important and useful instrument in electro-physiological researches. As we shall hereafter have occasion to refer to it, we take this opportunity of describing it. The preparation called by Matteucci "*the frog prepared according to Galvani's method,*" (la grenouille preparée à la manière de Galvani) consists of the skinned hind extremities of a vivacious frog, attached to a small portion of the spine by the sciatic or lumbar nerves only. The *frog galvanoscope* (grenouille galvanoscopique) on the other hand, consists of one skinned hind extremity of the frog, to which is attached a long nervous filament (the sciatic nerve). The limb is introduced into a glass tube, from out of which the nerve is allowed to be pendent.

"To employ this galvanoscope we take hold of the tube by the opposite extremity to that containing the frog's limb, and cause the nerve, which projects from the tube, to touch two points of the electro-motor element which we wish to examine. If the nervous filament be traversed by an electric current, the leg instantly contracts. If it be suspected that the direct contact of the nerve with the points of the electro-motor element might irritate the nerve, independently of the electric current, the suspicion may be readily obviated by placing two pieces of paper moistened with water on the two points of the electro-motor element, and afterwards closing the circuit by touching the two papers with the nervous filament.

"The frog used in this way, which I shall hereafter call the *frog galvanoscope,* is undoubtedly the most sensible apparatus we possess, if we renew it from time to time. The same instrument is also very accurate in its indications, if the nerve have been carefully dissected out so as to leave no matter adherent to it, and if the circuit be closed by the nerve only; no portion of the muscle of the leg being comprehended in it. The frog galvanoscope indicates to us not only the existence of an electric current, but also, with a very high degree of probability, the direction of the current. When the frog is somewhat weakened, we almost constantly see the leg contract when the circuit is closed, and remain unmoved when the circuit is opened, provided the current is in the direction from the nerve to the leg. If the phenomena occur in an inverse order, it arises from the current passing in the opposite direction, that is, from the leg to the nerve. We have then only to close the circuit with the nerve, and to open it the instant after, to determine at which of the two times contraction takes place. And to confirm the indication of the first experiment, we should reverse the position of the nerve: and if the first result were accurate, we shall now find that the limb will contract at the time when, in the first experiment, it was unmoved; and will remain tranquil at the period when before it was contracted." 30.

3. *Of the Electric Current in the Muscles of Living or recently Killed Animals.*—We have now to notice what we conceive to be the most interesting and important part of Matteucci's work; viz. that which treats of the electric current which exists in the muscles of animals both during life and for a certain period after death.

But before we proceed to notice his statements on this point, we think it due to the memory of Galvani to observe, that the existence of the muscular current seems to have been first recognised by this celebrated

Bolognian professor. It is well known that he believed in the existence of an *animal electricity*, the principal reservoirs of which were the muscles. The nerves he considered to be conductors; as serving to convey the electricity from the interior to the surface of the muscle. The apparent homogeneity of muscular tissue did not divert him from his hypothesis, for he found something analogous, something to support his opinion in the tourmaline.

But to return to Matteucci:—

"There is a very simple and very easily-made experiment," says he, "which proves the existence of an electric current developed by connecting, by means of a conducting substance, the different points of a muscular mass belonging to a living or recently-killed animal. We use for this purpose the *frog galvanoscope;* the nerve of which is to be introduced into a wound made by cutting the muscle of a living animal. By proceeding in this way it often happens that the frog is convulsed. But if we conduct the experiment carefully, we readily perceive that in order to be successful we must touch, with two different parts of the nervous filament, two different points of the muscular mass. Thus by touching, with the extremity of the nerve of the frog galvanoscope, the bottom of the wound, and with another part of the same filament the edge of the wound, or the surface of the muscle, the frog is instantly convulsed. This evidently proves that an electric current really circulates in the nerve, inasmuch as (to obtain the effect) we must form an arc in which this same nerve is comprised. That this contraction of the frog can be excited by an electric current due to the different points of the muscle of the living animal, is likewise clearly proved when we consider that, whatever be the liquid or conducting body with which the two parts of the nerve are touched, contractions are never excited." 52.

We may here observe that when an electric current is made to traverse a nerve centrifugally, that is in the direction from the brain towards the distal extremities of the nerve, it is called a *direct* current; but when in the opposite direction or centripetally it is termed an *inverse* current (p. 197.)

In every case contraction of the frog galvanometer is induced when the outer and inner surface of a muscle is connected by the nerve of the frog's leg. The experiment succeeds in fishes, reptiles, birds, and mammals; but the intensity of the current varies in different animals. The muscles of cold-blooded animals continue for several hours to give signs of the current.

If the *galvanometer* be substituted for the frog galvanoscope, deflection of the needle is obtained: thus satisfactorily proving that there is an actual electric current developed in the muscle, and that the effect on the frog galvanoscope is not due to any other cause. Moreover, this current exists in living, as well as in recently-killed, animals, when the circuit is closed between the interior and surface of a muscle. We think it unnecessary to describe all the precautions used by Matteucci to avoid various sources of fallacy; but we may observe that he has, in our opinion, satisfactorily proved that the current is not due to differences in the chemical nature of the fluids or to other adventitious circumstances; but really is a true muscular current. The direction of the current is, in every case, from the interior to the surface of the muscle; or, to speak more generally, from the interior of a muscle to any conducting body whatever which communicates with the surface of the muscle. Or we may say that the interior of the muscle is negative to the surface of the muscle; or that the surface of the muscle is positive to the interior.

By using a series of portions of muscles cut from recently-killed animals we form a *pile* or *battery*. In this way we may obtain a *frog battery*, an *eel battery*, a *pigeon battery*, &c.

To make a *frog battery* we take a number of skinned hind extremities of frogs. We cut off the legs at the knee-joint, carefully avoiding to injure the muscles of the thigh. Then divide the thighs transversely at about their middle. In this way we obtain conical-shaped pieces or half thighs. These are to be placed on an insulating tray (varnished wood) in which are two small saucer-shaped depressions or hollows capable of holding one or two drachms of water. These we shall call water-cups. A row or series of half-thighs is now formed on the tray, in such a way that the outer portion of each muscular mass (that is, the knee-end or smaller extremity of the half-thigh) shall be opposed to, and in contact with, the internal surface of the muscular mass (the cut surface of the half-thighs). One end of the row is in communication with one water-cup, the other end, with the other water-cup. Thus these cups, or rather the water which they contain, become the working poles of the battery, and they may be connected with the frog galvanoscope or the galvanometer, by means of platinum wire or moistened paper. A pile of twelve or fourteen pieces, or even less than this number, produces very marked and distinct effects on the galvanometer. In this pile the current passes from the exterior of one half-thigh to the interior of the second one, and from this to the surface of the same one, and thus to the interior of the third, and so on.

An *eel battery* is made with sections of the tail-end of the animal so disposed that the surface of one piece is in contact with the internal portion of the second piece, and so on for the whole series.

A *pigeon battery* is formed with slices of the pectoral muscles placed so that the outside of one slice is opposed to the inside of the next slice, and so on. A series of eight slices produces very distinct effects.

From a very elaborate series of experiments, which our space does not permit us to describe, Matteucci has drawn the following inferences:

"1st. That the intensity of the muscular electric current varies, in cold-blooded animals, proportionately to the temperature of the medium in which they have lived during a certain period.

"2ndly. That the duration of the current after death is shorter in proportion as the animal is more elevated in the scale of beings.

"3rdly. That the intensity of the muscular current varies with the degree of nutrition of the muscle, being greatest in muscles which are engorged with blood or which are inflamed.

"4thly. That this current is altogether independent of the integrity of the motor and sensorial nervous system, and of the activity of this system.

"5thly. That the influence of narcotic poisons on this current is either nothing or very feeble.

"6thly. That of different gaseous poisons, sulphuretted hydrogen is the only one which diminishes, in a remarkable manner, the intensity of the muscular current.

"7thly. That the direction of the muscular current is the same in all cases." 83.

What is the probable origin of the muscular current which is thus indubitably proved to exist? The experiments which we have detailed, neither prove the existence of free electricity in living animals, nor that electricity circulates in the nervous filaments. Matteucci has detected a current in muscular masses, without the integrity of the nervous system; and even when this system, on being irritated, ceased to produce contractions. The electric current appears whenever the circuit is completed between the outer and inner part of the muscles; and it is probable, therefore, that the structure and functions of these two parts differ.

As the signs of the current cease after a certain interval (which is greater in the inferior animals) subsequent to death, we may fairly conclude—

"That the organic disposition which constitutes the living muscular fibre, is as necessary as the action, whatever it may be, which maintains it in that state. This is furthermore confirmed by the influence exercised on the muscular current by the circulation of blood, by the redness of the muscular masses, by their condition of inflammation, &c." 124.

It is natural then to suppose that the nutrition of muscles and of other parts develops electricity. We know that the oxygen of arterial blood influences every part of the animal economy, that every part of the organism is incessantly undergoing a process of renewal, and that in every part this renewal is attended with a process analogous to combustion, by which carbonic acid is developed and heat extricated.

Matteucci compares the process by which the muscular current is generated to an ordinary voltaic apparatus. The oxidable element (*e. g.* the zinc) represents the fibre of the muscle; the acidulous liquid used to excite a voltaic apparatus is represented, in the animal, by the arterial blood; and, lastly, the non-oxidable element (*e. g.* the platinum or copper) is the analogue of the surface of the muscle, or of any other conductor not consisting of muscular fibre.

To explain more clearly Matteucci's hypothesis, we have drawn up the following diagram, denoting by the arrows, the direction which in each case the current follows:

| | | | |
|---|---|---|---|
| ↓ | Oxidable element (zinc) represents the | Fibre of the muscle. | ↓ |
| | Acidulated liquid . . . . . . . . | Arterial blood. | |
| | Non-oxidable element (platinum) . . | Surface of the muscle. | |

The nervous system may act, in the production of the muscular current, in two ways: 1st, as an imperfect conductor, which constitutes part of the circuit, and which is not the source of the electricity. This first function is entirely physical. 2ndly. It may also act by maintaining the cause which disengages the electricity; for nutrition is effected under the influence of this system. But as by cutting a nerve we cannot instantly arrest the chemical action attendant on nutrition, which is going on in the part supplied by the nerve, so division of the nerve is not immediately followed by a cessation of the muscular current.

We have thus endeavoured to give, as concisely as we could, Matteucci's hypothesis of the origin of the muscular current, and we proceed now to another topic.

4. *Of the Electric Current peculiar to the Frog.*—Besides the muscular current, another electrical current has been detected in the frog; and as

Matteucci has hitherto failed to recognise it in any other animal, he calls it the "*Courant propre de la Grenouille,*" or, as we have rendered it, the "*Current peculiar to the Frog.*" Its direction is invariably from the feet towards the head of the animal. Some of the phenomena dependent on it were successively recognized by Galvani, Humboldt, Valli, and Nobili.

"Nobili prepared the frog according to Galvani's method [which we have already described]. The preparation was then placed in such a position, that while the lumbar nerves were immersed in water in one cup, the legs were contained in the water of another cup. By plunging the two plates of the galvanometer into the water of these cups the circuit was closed. * * * * * * In this way Nobili obtained a deflection of the needle, to the extent of 5°, 10°, 15°, or more degrees. * * * * * * If the frog be robust, and have been quickly prepared, we see, at the moment of closing the circuit with the galvanometer, the limbs contract." 84.

A *battery* or *pile* may be formed by arranging a series of these preparations, the feet of one frog being in contact with the lumbar nerves of the next and so on. Or we may compose a pile, resembling Volta's *couronne des tasses*, with a series of cups, as above described. However formed, the direction of the current is constant, namely, in each frog, from the legs to the nerve, or rather from the feet towards the head.

The phenomena dependent on this current may be produced in a great variety of ways, for a description of which we must refer our readers to Matteucci's work. The current itself may be detected in three ways; by the galvanometer (as above described), by the frog galvanoscope, and by the contractions of the limbs of the preparation itself. Matteucci thus describes this last mode of proceeding:

"I remove the skin from the legs of a living frog, and cut the preparation longitudinally in the pelvic region, so as to expose the lumbar nerves. Then, by bending the legs we can bring them in contact with the lumbar nerves, and at that instant the contractions are observed." 87.

Matteucci has sought in vain for this current in other animals, although he has never failed to detect in them the muscular current. In salamanders, eels, and tortoises, he could find no current analogous to that found in the frog. He has, therefore, assumed that it is peculiar to this animal, and hence the name, which he has given it, of "*Courant propre de la Grenouille.*"

But against such an assumption we beg to enter our protest; because it is in opposition to every thing which we at present know respecting the organization and physiological relations of animals. It is with great diffidence that we venture to differ from Matteucci on any point of electro-physiology; but we cannot for a moment admit the probability of the frog possessing a peculiar electric current, unendowed as this animal is with any peculiar organs or electric apparatus. And on what grounds, it may be asked, is such an assumption made? Merely on negative evidence. Hitherto the author has not succeeded in detecting an analogous current in other animals, and he has, therefore, concluded (too hastily in our opinion) that it is peculiar, or proper, to the frog. We, on the other hand, confidently believe that, whatever currents may be detected in the frog, the same will be found to exist, in some degree of intensity, in other animals.

What is the origin of the *courant propre?* On this point nothing satisfactory is known. Some have regarded it as a *thermo-electric* current, dependent on the unequal temperature of the muscle and nerve, and arising from differences of evaporation. Others have considered it as an *electro-chemical* current; the leg being assumed to be charged with an alkali or with salts, and the thigh or lumbar nerve with some acid or weaker saline solution. Both these hypotheses Matteucci very properly rejects as untenable; and he observes that, if we refer the " courant propre " to the same origin as that assigned for the muscular current, it is necessary to assume that, the tendinous surface which composes the greater part of the frog's leg represents the interior of the muscle.

5. *Of a Physiological Phenomenon produced by a Muscle in Contraction.* —The phenomenon which Matteucci has treated of under the above title is a novel and highly curious one, which might be more expressively termed an instance of " electric sympathy." If the muscles of a frog prepared according to Galvani's method, or of a rabbit's thigh, be made to contract (by a current of electricity transmitted through the nerves or by any other method), a series of frog's legs may be made to contract simultaneously, (by sympathy, it would almost appear) provided the protruding nerves of each of the legs be laid across the muscles of the thigh of the rabbit or the first frog. The sympathetic movements do not occur if either a thin insulating plate, or a piece of gold-leaf be interposed between the contracting muscles and the nerves of the other legs.

Becquerel thinks these facts can only be explained by admitting that when a muscle contracts an electric discharge occurs; and that the electricity traversing the nerve of the frog's leg, occasions this also to contract. But if the electric current be prevented from entering the nerve, either by an interposed insulating plate, or by a better conductor, as gold-leaf, which conveys the current in another direction, the sympathetic contraction does not then take place. Perhaps the electric discharge is the muscular current whose intensity may become augmented by the contraction of the muscle.

6. *Of the Effects of the Electric Current on the Nerves.*—(A.) *On the Motor and Mixed Nerves.*—The effects of an electric current on the nerves vary at different periods after the death of the animal. Nobili fancied that he could distinguish five stages in the vitality of the nerve; but Matteucci admits only two. In the first of these, muscular contractions are produced, both at the commencement and at the interruption or cessation of the current: but in the second period they are observed in one case only, viz. either at the commencement or at the interruption of the current. In the case of the *mixed* nerves, (by which we mean nerves which are both sensitive and motor, and which, therefore, have double roots,) the contractions are observed *at the commencement of the direct current, and at the cessation of the inverse current.* But, with the *motor* nerves, the phenomena are reversed; for the contractions only occur *at the commencement of the inverse current, and at the interruption of the direct current.*

" The anterior spinal root," observe MM. Longet and Matteucci, " has been subjected to both *direct and inverse* galvanic currents in the four following conditions: 1st, the anterior and corresponding posterior root being untouched; 2ndly, both being divided; 3rdly, the posterior being untouched, the anterior divided; 4thly, the posterior being divided, the anterior being untouched.

"In every case, the contractions of the muscle or muscles animated by the anterior root on which the experiment was made, were manifested at first confusedly both at the commencement and at the cessation of the current, whatever its direction might be; but, after a certain interval, (which was somewhat longer when the anterior root was adherent to the medulla,) the effects became evident and durable: the contractions took place only *at the commencement of the inverse current, and at the interruption of the direct current.*"

Now we beg the especial attention of our readers to these curious results; which, to render more intelligible, we have thrown into a tabular form:

| | | Effects on *Motor Nerves.* | Effects on *Mixed Nerves.* |
|---|---|---|---|
| Direct or centrifugal current | Commencement.... | no effect. | Contraction. |
| | Interruption ...... | contraction. | No effect. |
| Inverse or centripetal current | Commencement.... | contraction. | No effect. |
| | Interruption ...... | no effect. | Contraction. |

A slight inspection of this table shews that the effects produced by the commencement of the direct current are identical with those caused by the interruption of the inverse current; and, *vice versâ,* those which the interruption of the direct current gives rise to, are identical with such as result from the commencement of the inverse current.

Not the least curious part of these phenomena is the opposition which appears to exist between the direction of the current and the direction of the action of the motor nerve. The latter is centrifugal, the former centripetal. The motor influence is propagated along the motor nerve in a centrifugal direction only; that is, in a direction from the nervous centres towards the distal extremities of the nerve. But in order to make manifest this influence by an electric current, we are obliged to cause this to traverse the nerve centripetally, that is, in a direction from the distal extremities of the nerve towards the nervous centres.

These facts appear to us to establish a great similarity between the effects of electric currents on the nerves, and those of *volta-electric induction* discovered by Mr. Faraday. It is well known that an electric current will induce another electric current in a neighbouring conductor; but the secondary or induced current takes place only at the moment of making or breaking the voltaic circuit. In the first case (that of making the circuit) the direction of the induced current is *opposite* to that of the primary or inducing current; while, in the latter case (that of breaking the circuit), the direction of the induced and inducing currents is the *same.* The secondary or induced current is only momentary. It does not exist during the continuance of the contact, but only at the moment of either making or breaking it.

If we were to assume that the physiological effect (*i. e.* the muscular contraction) was the immediate effect of a secondary or induced current, and not of the primary or inducing one, we should then find that the direction of the current and the direction of the action of the motor nerve would be identical; that is, both would be centrifugal.

We shall again resort to a table to illustrate our meaning.

| | | *Characters of the Secondary or Induced Current.* | *Effect on a Motor Nerve.* |
|---|---|---|---|
| 1. Direct or centrifugal Current induces | On making contact | an inverse or centripetal current | none. |
| | During contact .... | none | none. |
| | On breaking contact | a direct or centrifugal current | muscular contract. |
| 2. Inverse or centripetal Current induces | On making contact | a direct or centrifugal current | muscular contract. |
| | During contact .... | none.... | none. |
| | On breaking contact | an inverse or centripetal current | none. |

On this assumption we can easily account for a fact before stated, namely, that the effects produced by the commencement of the direct current are identical with those caused by the interruption of the inverse current, and *vice versâ*. Moreover, this assumption agrees with another fact, namely, that during the closure of the circuit or continuance of the contact, no contractions are produced; for, as will be seen by reference to the foregoing table, no secondary current exists at this period.

But if the muscular contractions, which result from the commencement or interruption of an electric current through the nerve, be due to a secondary or induced current, what structures, it may be asked, do the primary and secondary currents respectively traverse? To this question we are unable to give any definite reply. But, for the purpose of illustrating our position, let us assume that the primary current traverses the nerve coats, the secondary one, the primitive nervous fibres; and we shall then have the requisite conditions.

The complete opposition of effect produced by the electric current on motor and mixed nerves, led MM. Longet and Matteucci to repeat their experiments on various animals; namely, on the horse, the dog, the rabbit, and the frog. The results, however, were constant and invariable.

" But to produce them with success in the frog, it is indispensable (on account of the shortness of the roots, and of the extreme facility with which galvanic excitation is transmitted beyond the intervertebral ganglion, and, consequently, to the mixed spinal nerve) to take certain precautions which, although very simple, were only discovered by us after repeated experiments. After having separated the medulla from the encephalon, and opened the spinal canal from the abdomen, we insinuate pieces of varnished taffeta beneath the anterior lumbar roots, which are left adherent to a sufficient length of the spinal marrow. Then, having cut all the lumbar nerves of the opposite side, we apply the extremity of one reophorus (*pole*) on the anterior part of the medulla, and the extremity of the other on the anterior root. In this case the effects are soon manifested in as marked a manner as in the dog, that is to say, the contractions of the abdominal member are observed in two cases only; at the commencement of the inverse, and at the interruption of the direct current. But if, applying the two reophori (*poles*) on the anterior root itself, we approach the intervertebral ganglion, and the excitation be thereby transmitted to the mixed nerve situated immediately beneath this ganglion, the phenomena will seem to be reversed, and to appear to be such as occur with nerves which have not, as the anterior roots, an exclusively centrifugal action. It is a fact worthy of notice, that, by continuing to pass a current through the divided anterior roots (of the horse, dog, &c.), the muscular contractions, excited by the commencement of the inverse current, continue for a much longer time than those produced by the cessation of the direct current."

*Mode of distinguishing the Motor and Mixed Nerves.*—The experiments which have been devised and practised for ascertaining the functions of the nerves are the most cruel that can be imagined; while the results thereby obtained have frequently been unconvincing or uncertain. Every friend of humanity, therefore, will rejoice to find that in electricity we have an agent capable of distinguishing the motor and mixed nerves after the death of the animal. Alluding to the different and remarkable action of electric currents on nerves, either exclusively motor, or both motor and sensitive, MM. Longet and Matteucci observe that this appears to them "to constitute a sure means of distinguishing these nerves from each other, and may consequently serve to elucidate a question which has hitherto divided physiologists, namely, whether or not there are any nerves which are mixed at their origin."

(B.) *Effect of the Electric Current on Sensitive Nerves.*—MM. Longet and Matteucci, in another part of their memoir, describe the effect of electricity on the posterior or sensitive roots of the spinal cord. When these were separated from the cord no contractions were excited by the electrical current whatever be its direction.

"But when they were still adherent to the medulla, they *always* gave rise to convulsive shocks *on closing the circuit,* whether the current was inverse or direct. It was evident, however, that these convulsions were entirely due to a reflex action on the anterior roots; for, when the latter were cut across, all contraction instantly ceased."

*New Proof of the Functions of the Anterior Columns of the Spinal Cord.*—Another interesting point, which the researches of MM. Longet and Matteucci have elucidated, is the function of the anterior columns of the spinal cord, which are shown to possess an exclusively motor function; a fact which had been ascertained by physiologists by other modes of investigation; but which is thus singularly confirmed by the influence of electrical currents.

"Our experiment on the anterior white fasciculi of the spinal marrow, made on dogs, rabbits, frogs, and the common ringed snake (*Coluber Natrix),* have demonstrated that these fasciculi are affected by the *direct* and *inverse* currents in the manner of the anterior roots: a new proof of the exclusively motor function of the anterior white part of the cord."

*The posterior Columns of the Cord are entirely devoid of a Motor Function.*—MM. Longet and Matteucci's experiments confirm the common opinion of physiologists that, while the anterior columns of the cord are exclusively motor, the posterior columns are exclusively sensitive. For they found that, when all *reflex action* in the caudal extremity of the divided medulla of a dog had disappeared, "the (electric) stimulation of the posterior fasciculi never gave rise to the least muscular contraction, whatever was the direction of the current."

So that Bellingeri's groundless assumption of the motor function of all the white substance of the cord (the anterior columns being supposed to supply the flexor,—the posterior columns the extensor muscles) is thus satisfactorily disproved.

*Function of the Grey Substance of the Spinal Cord.*—With regard to the function of the internal grey portion of the spinal cord, MM. Longet and Matteucci observe that,—

"Some German physiologists having recently regarded the grey matter of the spinal cord as indispensable to the transmission of impressions and of the principle of voluntary movements, we think it proper to state that, in the dog, we have constantly found it insensible and inapt for producing convulsive shocks, under the influence of electricity and of mechanical irritants; and that its destruction, for as great a length as possible, by the aid of a stiletto, did not in the least modify the sensibility of the posterior medullary fasciculi or the excitability of the anterior ones."

(C.) *Conducting Power of the Nerves, &c.*—Matteucci infers, from experiments expressly made to determine the conducting power of different parts of animals, that "the conducting power of muscle may be assumed to be four times greater than that of the cerebral and nervous substances, which do not sensibly differ from each other in this respect." But he afterwards states that, in most of his experiments, the nerve appeared to conduct somewhat better than the cerebral substance.

(D.) *Circumstances which modify the Action of the Electric Current on the Nerves.*—Matteucci notices three, viz. Voltain alternatives, ligature of nerve, and poisons.

*a. Of the Voltain Alternatives.*—The discovery of the modification of the action of the electric current on the nerves, by the passage of the current itself through the nerves, was made by Volta.

"The current which traverses a motor nerve of a living or recently-killed animal, and which continues to pass in the nerve during a certain period, modifies the excitability so as to render the nerve insensible to the passage of the current, so long as it moves in the same direction. But the excitability of the nerve re-appears when the current is made to pass in the opposite direction. * * * * * So that at every change in the direction of the current, the limb, which previously contracted only when we close the circuit, becomes now capable of contracting at the interruption of the same circuit."

Matteucci found that the direct current destroyed the excitability of the nerve much more speedily than the inverse current. It also appears that a continued current less speedily exhausts the excitability of the nerve than a current alternately interrupted and renewed at very short intervals of time.

*β. Ligature of the Nerve.*—A ligature, it is well known, interrupts the passage of the nervous influence: how does it affect the electric current? It is commonly believed, even by some of the best writers on physiology, that the ligature of the nerve does not prevent the action of an electric current acting above the ligature. Matteucci, however, has shown this opinion to be erroneous; for, when complete insulation is adopted, the ligature of the nerve as completely interrupts the action of electricity as of any other stimulant.

*γ. Poisons.*—On this point Matteucci observes that he has found, "that *carbonic acid gas, nitrogen,* and *chlorine* do not weaken the excitability of the nerve submitted to the electric current; and the same may be said of *sulphuretted hydrogen.* One of the poisons which has the greatest power of enfeebling the excitability of the nerve is *hydrocyanic acid.*"

But if, when the nerves of a frog (poisoned by the latter substance) are no longer susceptible of the influence of a current transmitted through them, we pass the current through the muscles, contractions again appear, which, therefore, must be due to the action of the current on the muscles themselves. Death by an electric discharge destroys almost entirely the excitability of the nerve submitted to the electric current; but the muscles of frogs, who have been thus killed, retain their irritability; since the electric current again excites their contraction. In poisoning by solutions of extract of *opium* or of *nux vomica,* three stages or periods are distinguishable. In the *first,* the animal is in a state of super-excitation, and the feeblest contraction is sufficient to agitate the entire animal. In this stage the nerve is very excitable, and the current produces contractions, both at its commencement and cessation. In the *second* stage, tetanic convulsions are produced. If a frog, in this tetanic state, be submitted to the action of electricity, we perceive a slight shock at the commencement of the current; but after the circuit is closed, the tension of the muscles ceases, and relaxation takes place. Matteucci has observed a difference between the action of the direct current and that of the inverse one: the current directed from the feet to the head excites at the commencement a shock, which is weaker than that produced at the commencement of the direct current. In the *third,* or last stage, all tetanic movements have ceased; and then there is either no excitability of the nerve, or scarcely any; but the muscular fibre still retains its power of contraction when submitted to the electric current.

7. *Therapeutical Uses of the Electrical Current.*—We have now arrived at the last division of our subject, that which relates to the medicinal employment of electricity. Hitherto practitioners have, for the most part, prescribed this agent in certain diseases, as a last resource, when other remedies, in which more confidence is very properly placed, have failed. It is employed empirically: no very satisfactory principles, for the guidance of those who use it, having been ascertained. Let us see then, what are the practical conclusions which may be fairly drawn from Matteucci's electro-physiological researches.

In the first place, what are the diseases for the relief of which electricity deserves to be tried? Matteucci's researches lead him to believe that *paralysis*, either partial or total, of motion, of sensibility, or of both these functions, are the maladies for the alleviation of which the electric current deserves to be tried. He has also proposed it in *tetanus*. As to its employment *for the purpose of destroying the opacity of the crystalline lens*, as lately proposed, Matteucci candidly states that he thinks it is more calculated to make a cataract than to cure one. Nor does he think it probable that *urinary calculi* can be got rid of by electrolysis.

In the application of electricity to the treatment of paralysis and tetanus, it is necessary to bear in mind two electro-physiological facts. The *first* is, that an electric current, if transmitted through a nerve for a certain period of time, destroys the sensibility of nerve, or, in other words, paralyses it. If allowed to remain in repose, the nerve, after a certain interval, receives its excitability. But it has been ascertained by Matteucci that the excitability may be restored in a much shorter period by passing a second current through the nerve in the reverse direction. The *second* fact to be borne in mind is, that if the nerves of a living animal be submitted to the passage of the electric current, renewed at short intervals, tetanic contractions are excited; and if the experiment be continued for some time the nerves entirely lose their excitability.

"These are the facts," says Matteucci, "which, independently of all theory or hypothesis, should guide us in the therapeutical application of the electrical current to palsies. We may, in fact, admit that, in some cases of paralysis, the nerves of the affected limb are in a condition similar to that produced by the continued passage of an electric current. We have seen that, to restore the excitability to a nerve which had been deprived of it by an electric current, it is requisite to conduct the current in the opposite direction. Hence to cure the paralysis, the current should be passed in a contrary direction to that which has produced it. In a paralysis of motion the inverse current should be employed; while, on the contrary, in a paralysis of sensation, the direct current should be used. In a case of complete paralysis [that is of both motion and sensation] there is no reason to induce us to prefer the one current to the other.

"Theory also teaches us a rule in its application: never to continue the passage of the current too long, lest we augment the disease we wish to cure. The more intense the current the shorter should be its duration; and as we have seen that the passage of the electric current in the nerves, repeated at short intervals of time, considerably enfeebles their sensibility, when continued for a long time, we must take care and not pass from one extreme to another. Theory advises us to apply the electric current of an intensity which should vary with the degree of the malady, and continue its passage for two or three minutes at intervals of some seconds. After these two or three minutes, during which we shall have communicated from twenty to thirty shocks, we should leave the patient at rest for some time, and then renew the treatment."

In 1838, Matteucci published a case of tetanus in which electricity was tried. During the passage of the current the convulsions ceased, and the patient opened his mouth. But the amelioration was only temporary, the disease being occasioned by the presence of some foreign bodies in the muscles of the leg.

He has shown that the prolonged passage of the uninterrupted current through a limb causes paralysis of the part. Hence he was led to suppose that the tetanic condition of a limb may be removed by inducing paralysis. And he found, in support of his notion, that frogs rendered tetanic by nux vomica, lost this condition under the influence of an electric current.

In concluding our notice of M. Matteucci's electro-physiological researches, we beg to observe that we have considered it expedient to omit all notice of his interesting researches respecting the electrical phenomena of the *Torpedo* and *Gymnotus*, as likewise Savi's elaborate anatomical description of the nervous system, and of the electric organ of the *Torpedo*. It would have been impossible to have given an intelligible notice of these subjects in a reasonable compass, and we have preferred occupying our pages with those details which have a more immediate relation to human physiology, well knowing that those persons who take much interest in comparative anatomy and physiology will procure Matteucci's work.

We have already expressed our opinion of the great scientific value and importance of Matteucci's researches. His book is illustrated by six plates, three of which refer to Savi's Anatomy of Torpedo. The Memoir of MM. Longet and Matteucci is very short, but is a highly interesting one; being devoted to an account of their discoveries of the different effects of electric currents on the different kinds of nerves. Of these discoveries we have given a tolerably full notice. This memoir, we perceive, has been reprinted in De la Rives' *Archives de l'Ectricité*, published in October last.

