# Errata

**Published:** 1845-01

**Authors:** 


					d m t0P>J?r induction, read introduction,
rage d-0, line 13, for receives, read recovers.

				

